# I dream of socializing, sports, and serenity: Imagining a positive future‐vaccinated self is associated with better attitudes toward COVID‐19 vaccination

**DOI:** 10.1111/jasp.12909

**Published:** 2022-07-27

**Authors:** Genavee Brown, Anne‐Laure de Place

**Affiliations:** ^1^ Department of Psychology Northumbria University Newcastle upon Tyne UK; ^2^ Laboratoire Parisien de Psychologie Sociale (LAPPS) Université Paris 8 Vincennes ‐ Saint‐Denis Saint‐Denis France

## Abstract

In the context of the COVID‐19 pandemic, governments are attempting to vaccinate a large proportion of their adult population against the virus. While many people hurried to receive the vaccine, vaccination rates then started stagnating and governments are searching for solutions to motivate remaining citizens to receive the vaccine. Previous studies show that imagining oneself in the future can motivate health prevention behaviors, but our study is the first to use a future selves paradigm to study vaccination motivators. In two mixed methods studies we examine the effects of imagining of a future‐vaccinated self (FVS) on vaccine attitudes, where participants were asked to think about what their life would be like once they had received the COVID‐19 vaccine. In Study 1 (*n* = 114), we coded the most important categories of FVS. Several FVS were identified and related to increased social and leisure activities, reduced negative emotion and societal constraints, possible side effects of the vaccine, and societal changes. In Study 2 (*n* = 113), we used a 2 × 2 design in which participants' reflections on their FVS were guided or open and visualized from a first‐ or third‐person perspective. The guided condition produced greater acceptance of the vaccine, and the first‐person perspective produced greater behavioral intentions to be vaccinated. We discuss the effectiveness of future selves interventions for promoting vaccination in different societal contexts.

## 1 INTRODUCTION

A TV advertisement begins with a man walking into a locker room, gym bag in hand. All his teammates are there; in their excitement to be reunited, they throw themselves at him and Johnny Halliday's *Que je t'aime (How I love you)* plays over these touching images. The shot cuts to the playing field, where the friendly scrum turns into a virile tackle in front of invested supporters, then another cut reveals that the man was in fact daydreaming while getting his vaccine. A nurse leans over him: “Sir, are you OK? It didn't hurt?” and he answers, with feeling: “Hurt? Oh no, in fact it feels great!.” This television advertisement, launched by the French Ministry of Solidarities and Health in May 2021 as part of a national campaign to encourage vaccination against COVID‐19, is designed to evoke an individual's future or *possible* self (Markus & Nurius, 1986) to motivate them to get vaccinated. It demonstrates the idea that imagining a future self can motivate current behavior (Markus & Nurius, 1986).

In the current set of two studies, conducted in France, we aim to explore what types of future selves might motivate vaccination. In the first study, we asked participants to tell us about what they thought their lives would be like after they had been vaccinated. We then qualitatively analyzed their responses. In the second study, we built on results from the first study aiming to manipulate the ease with which participants could imagine their future self by guiding their reflection and asking them to imagine it from a first‐ or third‐person perspective. In both studies, we measure vaccine acceptance and intention to receive the vaccine. We then test the relationship between imagining a future‐vaccinated self (FVS) and these outcome variables. We hope that these studies can provide guidance to authorities developing campaigns to help motivate vaccination against COVID‐19.

## 2 FUTURE SELVES AND HEALTH BEHAVIOR

Appealing to the imagination of individuals and their ability to project themselves into a better future could be one option to motivate vaccination even when individuals do not perceive COVID‐19 as an important risk to their health. Possible selves or future selves, defined as mental images of what one would like to become or fear becoming in the future, have indeed been found to energize and organize individuals' behaviors towards a desired future or away from a worrying one (Markus & Nurius, 1986; Ruvolo & Markus, 1992). While Markus and Nurius (1986) originally wrote about “possible selves,” the expression “future selves” is now used indiscriminately in the literature (de Place & Brunot, 2018). To emphasize the fact that we will be talking specifically about possible selves related to a change in vaccination status, we will use the phrase “future vaccinated selves” (FVS) throughout the article. Possible future selves represent visions of the future that an individual deems possible for them, whether positive (“I hope I'll get my dream job”) or negative (“I fear I'll be out of work when I'm fifty”). A possible self is not a fantasy, or an abstract belief: It includes a mental image of oneself in the situation, a feeling of what one would experience should it happen (Cross & Markus, 1991; Erikson, 2007). For example, hoping to get your college degree is not a possible self, unless you imagine the way you'll behave and feel on graduation day.

To understand the motivational power of future selves, researchers have developed mental imagery procedures that manipulate the way a possible self is brought to mind (Ruvolo & Markus, 1992). Artificially creating a new future self is not the goal: these imagery procedures aim to make certain facets of an existing possible self more accessible, to determine how it impulses behavior. In their seminal study, Ruvolo and Markus (1992) showed the immediate motivational impact of imagining a positive or negative possible self on the completion of a difficult cognitive task. Since then, numerous studies have manipulated the salience of different possible selves to increase motivation and performance in other domains (e.g., academic; Landau et al., 2014; de Place & Brunot, 2020). Increasing the salience of FVS could therefore be a way to motivate individuals to get vaccinated.

As a matter of fact, the motivational impact of future possible selves has already been studied in the health domain, particularly with young people presenting no particular risk factors. In a review of 14 studies focused on the influence of possible selves on health‐related behaviors in adolescents, Corte et al. (2022) concluded that a high likelihood of achieving a desired possible self was often associated with lower levels of health‐risk behaviors and higher levels of health‐promoting behaviors. Several interventions to promote healthy activities among young people have relied on guided imagination of possible selves (e.g., Murru & Martin‐Ginis, 2010; Ouellette et al., 2005). In these studies, participants were asked to see themselves in the future as a healthy and athletic person or as someone unfit and in poor health. Compared to a control condition without any imagery, participants who had to reflect on a positive or negative future self had significantly increased their physical activity up to 8 weeks later.

To initiate a change in behavior, an adequate gap must be perceived between a possible future self and the actual self (Hoyle & Sherrill, 2006): imagining an unchanged future situation (“I'll still be overweight in ten years”) is unlikely to be motivating, as a is a future self that is unlikely to be obtained and therefore difficult to imagine (“I'll run a marathon next year even though I've never been a runner and don't know how to train”). Additionally, other characteristics of possible selves contribute to the motivational impact of these images of the future (for reviews see de Place & Brunot, 2018; or Oyserman & James, 2009). The first and most important one is the elaboration of the future self: the clearer, easier, more detailed, and narratively rich the vision is the greater its motivational influence (e.g., King & Raspin, 2004; Norman & Aron, 2003). With regard to vaccination against COVID‐19, being able to imagine a clear, detailed FVS might then lead to better attitudes towards vaccination than remaining in the dark about what could change postvaccination, or believing that it will not change anything to the current pandemic challenges.

One final characteristic which can impact the motivational power of the future self is the visual perspective with which it is imagined. Indeed, a future situation can be visualized in two possible ways: either in the first person, that is, through the participant's own eyes, or in the third person, that is, through the eyes of an observer of the situation (Nigro & Neisser, 1983). Nurra and Oyserman (2018) showed that visualizing a possible self in the first‐person perspective led to a greater feeling of connection between the present self and the future one. Although certain research found that imagining a positive future self in the third‐person perspective led to more motivation and behaviors to approach it (Libby et al., 2007; Vasquez & Buehler, 2007), a series of studies that manipulated health‐related possible selves concluded, on the contrary, that first person visualization had a greater impact on behavioral intention (Rennie et al., 2014). Further studies are necessary before a definitive conclusion can be drawn about the motivational impact of differing visual perspectives. In the case of COVID‐19 vaccination, the current state of research leads us to hypothesize that visualizing a FVS in the first person might lead to a higher intention to get vaccinated.

## 3 PRESENT RESEARCH

Our aim with this study is to understand what types of FVS participants might imagine, as we could not find any research on the motivations for COVID‐19 vaccination which were unrelated to risk (Li et al., 2021; Tran et al., 2021) or prosocial motivations for COVID‐19‐related health behaviors (Marinthe et al., 2022; Wentzell & Racila, 2021). Thus, we hoped to capture other visions of the future that might motivate vaccination, especially for people at low risk from COVID‐19. We also wanted to examine whether elaborate, easier to imagine, and vivid FVS would be related to better attitudes towards the COVID‐19 vaccine (higher acceptance and intention to receive the vaccine). Finally, we wanted to determine whether visualizing these FVS from the first‐person perspective would lead them to have a greater impact than FVS imagined in the third‐person perspective. All quantitative data, participant details, and supplementary analyses from both studies can be found on the OSF: https://osf.io/753k8/?view_only=6e0282da1fcf44688de45ae1e919cb27 along with preregistration documentation for Study 2: https://osf.io/qsx6y/?view_only=cdbe8d4ac11c4ac48be11f4075aa0a03.

## 4 STUDY 1

Study 1 was largely exploratory and was mainly concerned with understanding what types of future selves participants at low risk from COVID‐19 would imagine concerning their life once they had received both doses of the COVID‐19 vaccine. We created five open text boxes where participants could respond about anything they wished concerning their FVS. They were then asked several questions concerning the characteristics of this FVS and about their intention to receive the COVID‐19 vaccine and their acceptance (efficacy and risks) of this vaccine. We conducted a content analysis on participants' responses concerning their FVS and coded several recurring categories as well as the valence of their response.

### 4.1 Methods

#### 4.1.1 Participants

Participants were 114 people living in France (98 women, 14 men, 2 nonbinary or questioning, age: *M* = 24.10, *SD* = 8.16) recruited online via social media from January 16, 2021 to January 25, 2021. At this point, vaccine diffusion in France was only open to people over 75 years old (50 for medical personnel), those with risk factors and nursing home residents. As none of our participants fell into these categories, they had not received any doses of the COVID‐19 vaccine. We stopped data collection after 10 days because of the quickly changing nature of the vaccination campaign. A post hoc sensitivity analysis conducted on G*Power shows that our sample size allows us to detect correlations above *r* = .15.

#### 4.1.2 Procedure and materials

The Ethics Committee at Northumbria University approved this study before data collection began. Participants were recruited via Facebook groups, particularly French university student groups. Participants read an information sheet and were asked for consent before completing the online questionnaire.

4.1.2.1

*FVS imagery task*

Participants were asked to imagine a future in which they had been vaccinated against COVID‐19. They were encouraged to take 2–3 min to imagine themselves in this situation, then list the “five most important things” which would change in their life once they had received the COVID‐19 vaccine, when compared to their life today. Five open text boxes were provided for the answers.

4.1.3

*Characteristics of the future self*

Five questions were designed to measure characteristics associated with the FVS that may influence its motivational impact, namely the difficulty of imagining the future self, the vividness of the image created, the frequency with which participants thought about it, the probability that the future self would be realized and its perceived temporal distance from the present self. Answers for the first four questions were provided on a 7‐point Likert‐style scale (from 1 = *not at all probable* to 7 = *very probable* for the probability measure for instance). Temporal distance was measured by asking the participant to move a cursor on an axis going from *very close* to *very distant*.

4.1.4

*Vaccination intention*

Intentions regarding the COVID‐19 vaccine were measured by four items adapted from previous research (e.g., Kim & Nan, 2016; Liu et al., 2019). Two questions measured the intention to get vaccinated against COVID‐19 (e.g., “Do you intend to get the COVID‐19 vaccine?”) and two questions asked about the intention to recommend the vaccine to relatives (e.g., “If you had to give your close friends and family advice about having the vaccine, would you advise them to get the COVID‐19 vaccine?”). Answers were given on a 5‐point Likert‐style scale, from 1 = *definitely not* to 7 = *definitely*. Alpha for this scale was .93.

4.1.5

*Vaccine acceptance*

Acceptance of the COVID‐19 vaccine was measured by six items adapted from Nan et al. (2012). Three items measured perceived vaccine safety (e.g., “I worry about the short‐term side effects of the COVID‐19 vaccine”) and three measured perceived vaccine efficacy (e.g., “I believe the COVID‐19 vaccine is effective in preventing COVID‐19”). Participants answered on a 5‐point Likert‐style scale, from 1 = *strongly disagree* to 7 = strongly *agree*. Although the scale has two dimensions, they were highly correlated (*r* = .626, *p* < .001) and Cronbach's alpha = 0.90 for all items combined. Therefore, we chose to use all six items as one variable called vaccine acceptance.

4.1.6

*Demographics*

Demographics measures (age, gender, nationality, native language, vaccine status, and COVID‐19 experiences) concluded the study, followed by a debrief page that explained the purpose of the study to participants.

### 4.2 Results

#### 4.2.1 Qualitative results

Content analysis was used to describe the qualitative data obtained in the five open text boxes. In line with the exploratory approach of this first study, categories were derived from the data (vs. defined a priori). Two researchers read all the open‐ended answers and after a trial and error procedure agreed on 10 categories, which encompassed all the participants' answers. Each researcher then independently coded the whole database. Discrepancies in coding were very limited; they were resolved by discussion.

The 10 agreed‐upon categories and their prevalence in the participants' five FVS are presented in Table 1.

**Table 1:** Prevalence in percentages of the different FVS categories in the five open‐ended answers and overall occurrence of each category in percentages

Category	Definition	FVS1	FVS2	FVS3	FVS4	FVS5	Overall
1. Return to social life	*Reunion with friends and family, resuming face‐to‐face interactions*.	27.2	30.7	28.1	22.8	18.4	25.4
2. Resumption of leisure activities	*Return to bars, restaurants, or museums, resuming traveling and sport*.	22.8	26.3	26.3	24.6	23.7	24.7
3. Reduction of negative emotions	*End of the worrying and other negative emotions related to the pandemic*.	19.3	13.2	13.2	16.7	15.8	15.6
4. End of constraints	*End of the mask wearing and social distancing*.	10.5	7	6.1	7	6.1	7.3
5. Societal change	*Emergence of a new way of living by learning from the pandemic*.	0.9	5.3	5.3	3.5	9.6	4.3
6. Worries about the vaccine	*Worries about side‐effects of the vaccine, from illness to death*.	5.1	8.8	6.1	3.5	7	6.1
7. Negative societal change	*Concerns about enduring liberty restrictions and other negative societal consequences of the pandemic*.	2.6	2.6	4.4	6.1	4.4	4
8. No change	*No change expected after getting the vaccine*.	4.4	3.5	2.6	6.1	1.8	3.7
9. No vaccine/imagery refusal	*Refusal to get the vaccine (i.e., to complete the imagery task)*.	1.8	1.8	3.5	1.8	1.8	2.1
10. Other or no answer	*Absence of a categorizable answer*.	4.4	0.9	4.1	7.9	11.4	5.7

Abbreviation: FVS, future‐vaccinated selves.

4.2.2

*Return to social life*

This category represents about a quarter of all FVS mentioned by the participants. Many images revolve around reuniting with friends and families having fun as a group: “I can go out and have fun”; “see my friends more often”; “visit my family members.” In our predominantly student sample, the return to social life also largely involves resuming face‐to‐face classes: “I can go back to class”; “I can go back to study in person at university.”

4.2.3

*Resumption of leisure activities*

The second most often mentioned category overall, this category mainly includes the answers of participants who wish to resume their leisure activities in places closed during the pandemic, such as bars, museums, or cinemas: “I can visit bars”; “go to the movies”; “opening of the restaurants.” We note that many participants mention the possibility of traveling again (“I can go on vacation”), and that the resumption of indoor sports is also an important element for some of them (“I can do any kind of sport”). Finally, a typically French activity also appears in this category: demonstrations, that were made much more complicated by health constraints (“I will go to demonstrations more often”).

4.2.4

*Reduction of negative emotions*

This category is the third most frequently mentioned. Many FVS in this category tell of the end of the stress related to the possibility of getting sick: “I no longer fear covid,” “A weight is lifted from my shoulders,” “My mental health has greatly improved.” Feelings of anger or distrust are also expected to diminish: “I stop being angry at people's lack of responsibility,” “I am more relaxed at work, I feel safer,” “I am no longer paranoid.” Many of the emotions mentioned reveal a genuine prosocial attitude among the participants: the COVID‐19 vaccine would reassure them not only for themselves but also for their loved ones. They state for example: “I can go out without feeling guilty about taking covid home,” “I no longer feel irresponsible when I am meeting my friends.”

4.2.5

*End of specific constraints related to the management of the pandemic*

In this category, we grouped all answers related to the end of the constraints placed upon French people for health and safety reasons during the pandemic. Mentions of the end of mask wearing are numerous, which shows the symbolic importance of this restriction: “No more mask wearing,” “I can go out without a mask.” Other constraints were discussed, especially the end of social distancing, for instance “kissing and hugging people.” Finally, the notion that a “normal” life will return is present in the participants' answers; a definite desire to return to life as we knew it before the pandemic: “Life is normal again,” “Life has resumed its course.”

4.2.6

*Societal change*

Positive societal change, a new way of living brought upon by the pandemic, was also present in the minds of many participants. Their preoccupations were both individual (“A new awareness of what we really need,” “More pleasure in enjoying simple things like the freedom to come and go”) and collective, with massive changes imagined especially in favor of the environment (“Important measures taken for the climate,” “We pay more attention to the environment”).

4.2.7

*Worries about the vaccine*

Most of the answers in this category are from participants who are wondering about possible side effects of the COVID‐19 vaccine, without elaborating more on their fears: “I am afraid of possible consequences of the vaccine,” “Worries about the side effects.” Some went further: “Probable health problem,” “I have become sterile,” “I lose 30 years of life expectancy.” Some rare participants even evoked outright conspiratorial arguments: “The vaccine will have eradicated many people,” “Nanoparticles are directly in my body,” “The government can control my brain remotely.” Finally, four people expressed their conviction that the vaccine would kill them.

4.2.8

*Negative societal change*

Contrary to the “societal change” category presented above in which participants hoped for a new global awareness and a positive lifestyle change, the answers of this category are decidedly negative. Many expressed concerns about future government decisions to address the pandemic and the societal divides it could bring: “Distrust in government has increased,” “Individuals must have an immunization card with them at all times,” “A divide is created between the vaccinated and the others.” They worry about the recurrence of pandemics (“a new pandemic threatens as the permafrost melts”) but also “global wars” and “economic crises” that will “alienate” and “put us down.” Some go as far as to say that “Everyone will have to save their own skin” and that “the only solution is to leave for another planet.”

4.2.9

*No change*

For the participants whose answers are coded in this category, there was no enthusiasm for the arrival of the COVID‐19 vaccine. Their FVS reflect no change from the current situation (“Nothing changes”), especially when it comes to health constraints: “Wearing a mask is still mandatory,” “Social distancing despite the vaccine.” As one participant said: “I don't feel better.”

4.2.10

*Vaccine refusal (refusal of the imagery task)*

Two participants repeatedly indicated that they would not get vaccinated (in the five text boxes). They were excluded from the subsequent quantitative analyses since they did not complete the imagery task that required them to project themselves after getting the COVID‐19 vaccine.

4.2.11

*Other or no answer*

This category was used to group answers that we did not understand or that amounted to an absence of answer (without being a clear refusal of the task). The number of answers included in this category increased from 3 to 11 for the fifth box.

#### 4.2.12 Quantitative results

To explore the relationships between our variables we ran bivariate correlations. We found that our dependent variables of intention to receive the COVID‐19 vaccine and acceptance of the COVID‐19 vaccine were strongly positively correlated. Most of the characteristics of FVS were correlated, suggesting that those people who had a clear picture of their future self had less difficulty imagining their future self, thought about it more frequently, thought it was more likely to happen, and imagined it as less distant temporally. Interestingly, although all the characteristics of FVS were moderately correlated, we noted that only difficulty and frequency of imagining the future self were positively correlated with both the intention to receive and acceptance of the vaccine. One final result of note is that the risk related to being infected with COVID‐19 and acceptance of and intention to receive the COVID‐19 vaccine are only weakly correlated in our sample. All bivariate correlations are reported in Table 2.

**Table 2:** Bivariate correlations between variables

Variables		1	2	3	4	5	6	7	8	9
1	Acceptance of vaccine	–								
2	Vaccination intention	0.839**	–							
3	Difficulty to imagine	0.338**	0.306**	–						
4	Vividness	0.107	0.168	0.482**	–					
5	Frequency	0.187*	0.286**	0.297**	0.474**	–				
6	Probability	0.126	0.123	0.282**	0.301**	0.118	–			
7	Temporal distance	0.027	0.126	−0.192*	−0.327**	−0.032	−0.556**	–		
8	Perceived risk	−0.178	−0.189*	0.057	0.008	−0.001	0.048	−0.061	–	
9	Age	−0.108	−0.033	−0.062	0.006	−0.006	0.174	−0.153	0.066	–

** *p* < .01.

* *p* < .05.

### 4.3 Discussion

The results of this first study provide interesting insight into how people view their FVS and what might motivate them to receive the COVID‐19 vaccine. Participants cited a return to socializing, engaging in leisure activities, a reduction of negative emotions, and an end to specific constraints as the most frequent positive outcomes related to receiving the vaccine. While the overwhelming majority of the FVS reported were positive (80%), some participants did indicate concern about the safety and efficacy of the COVID‐19 vaccine. We observed that the difficulty of imagining the future self played a role in participants' attitudes toward the COVID‐19 vaccine and that when they had a harder time imagining their FVS they had less positive views of the vaccine. Thus, in our second study we manipulate the difficulty of imagining the future self to test experimentally whether it could have an impact on attitudes toward the COVID‐19 vaccine.

## 5 STUDY 2: INTRODUCTION

The goal of Study 2 was to experimentally manipulate the difficulty of imagining a FVS. To see if we could make participants imagine a more detailed and elaborate FVS, a guided reflection task was introduced and compared to the open reflection task used in Study 1. As more elaborate visions of a future self are more motivating to attain this future self, we predict that people in the guided condition will have greater intention to receive the COVID‐19 vaccine and greater acceptance of the vaccine. Second, we manipulated the perspective (first vs. third) taken when imagining the future self. The few studies existing in the health domain show a more positive impact of first‐person imagery on health behaviors (Rennie et al., 2014) but further studies are needed to confirm this. We predict that imagining a FVS from the first‐person perspective will lead to greater acceptance and intention to receive the COVID‐19 vaccine.

### 5.1 Methods

#### 5.1.1 Participants

One hundred and thirteen participants living in France completed Study 2 (89 women, 22 men, 1 nonbinary person, and 1 unreported, age: *M* = 28.27, *SD* = 10.20). An apriori G*Power analysis suggested that to detect a small effect size, we would need a total sample of at least 108 participants, and we met this criteria (Faul et al., 2007). Participants were contacted through social media and completed the survey between March 12, 2021 and April 6, 2021. This was during a nationwide curfew when French citizens had to return home by 6 p.m. each evening. At this point the age threshold to get vaccinated when one had no specific risk factors was still 75 years old (it got lowered to 70 by March 25), and vaccination had been opened to all medical professionals.

#### 5.1.2 Procedure and materials

As in the first study, ethical approval was obtained from Northumbria University's Ethics committee. Participants were recruited from Facebook groups associated with French universities. They completed the questionnaire online after filing out a consent form.

5.1.3

*FVS imagery task*

The imagery task began as in Study 1 by asking participants what would change once they had been vaccinated. Perspective was then primed by the following instruction: “To best imagine this situation, you should visualize it in the first [third] person.” Participants in the first person condition then read the following sentence: “You see the events with what your own perspective would be, in other words you observe the environment with your own eyes.” Participants in the third person condition read: “You will see the events occurring from the perspective of an observer of the situation, in other words, you will see yourself in the same way you would see the environment, like seeing yourself in a movie.” These directions were adapted from previous works on future selves imagery perspective (e.g., Rennie et al., 2014). The presentation of the answer box was also manipulated to influence the elaboration of the FVS. For half the participants, a single open text box was provided, whereas for the other half, four text boxes were provided, labeled with the four most frequently occurring categories from Study 1: “social life,” “leisure activities,” “emotions,” and “everyday life” (a wording chosen to evoke the fourth category, “end of specific constraints related to the management of the pandemic”, without having to explicitly mention mask wearing or social distancing). Participants were thus randomly assigned to one of four experimental conditions: “imagery in first person, open reflection” (*n* = 27), “imagery in third person, open reflection” (*n* = 30), “imagery in first person, guided reflection” (*n* = 29), and “imagery in third person, guided reflection” (*n* = 27).

5.1.4

*All other measures*

The characteristics of the FVS (five items), vaccination intention (four items), COVID‐19 vaccine acceptance measures (six items), and sociodemographic measures were the same as in Study 1.

### 5.2 Results

#### 5.2.1 Qualitative analysis

5.2.2

*Responses in the guided conditions*

In the guided conditions, most participants correctly answered all four items, with two participants failing to understand one category, and a participant who answered incorrectly for three out of four categories and who was excluded from analyses.

Responses were similar to Study 1. There were two notable differences. First, the participants offered more detailed responses in the guided conditions. Second, in the emotions category, they tended to express an increase in positive emotions rather than the reduction of negative emotions.

5.2.3

*Responses in the open conditions*

For the open (nonguided) conditions, coding was performed by the two researchers independently, using the 10 categories derived from Study 1. Minor discrepancies were resolved by discussion; the categories developed in Study 1 fit the data in Study 2 well. Percentages of responses by condition and overall for the open condition are reported in Table 3. Due to the similarities between the responses in this study and Study 1 we have not discussed them in detail. Indeed, even the percentages of responses by category are very similar to Study 1. The only important change was that the fourth positive category, which included future thoughts about the end of constraints such as wearing a mask or respecting social distancing guidelines, was more frequent in Study 2 than in Study 1. This is probably because more participants spoke of their hope of the national curfew coming to an end, for instance: “Living without having my eyes glued to my watch and without being vigilant to the passing hour and calculating the time it will take me to get home.”

**Table 3:** Percent of FVS categories reported by condition

Category	First‐person open	Third‐person open	Total (all open responses)
1. Return to social life	27.4	18.2	23.1
2. Resumption of leisure activities	19.4	21.8	20.5
3. Reduction of negative emotions	9.7	12.7	11.1
4. End of constraints	21.0	9.1	15.4
5. Societal change	8.1	1.8	5.1
6. Worries about the vaccine	4.8	12.7	8.5
7. Negative societal change	1.6	9.1	5.1
8. No change	4.8	5.5	5.1
9. No vaccine/imagery refusal	1.6	7.3	4.3
10. Other or no answer	1.6	1.8	1.7

*Note*: Percentages are from a total of 62 categories cited by participants in first person open condition, 55 categories cited in third person open condition, and 117 categories in the two open conditions combined.

Abbreviation: FVS, future‐vaccinated selves.

5.2.4

*Valence profiles across all conditions*

Based on the qualitative analysis, we noted that some participants gave all‐positive, mixed (some negative, some positive), or all‐negative FVS and were thus classed into profile groups. As in Study 1, if a participant completely refused to imagine vaccination, we removed them from the analysis. In total, two participants were excluded from the open condition for this reason and one from the guided condition due to a refusal to answer the prompts. Thus, the total sample for quantitative analysis was 110 participants.

#### 5.2.5 Quantitative analyses

5.3

*Effects of the conditions on FVS characteristics*

To test the effectiveness of our experimental manipulation (guided vs. open and first vs. third perspective) we ran 2 × 2 analysis of variances (ANOVAs) on the number of words written, the number of categories used to describe FVS, and the difficulty reported in imagining their future selves. For the number of words, only the independent variable of guided versus open had a significant effect, *F*(1, 106) = 41.597, *p* < .001, partial *η* ^2^ = 0.282. Participants in the guided condition (*M* = 92.28, *SD* = 48.23) used more words than those in the open condition (*M* = 40.31, *SD* = 38.78). Perspective, *F*(1, 106) = 1.748, *p* = .185, partial *η* ^2^ = 0.017 and the interaction between perspective and guided versus open, *F*(1, 106) = 2.779, *p* = .098, partial *η* ^2^ = 0.026, did not have a significant effect on number of words written by participants.

For the number of categories, both perspective, *F*(1, 106) = 5.157, *p* = .025, partial *η* ^2^ = 0.046, and guided versus open, *F*(1, 106) = 121.432, *p* < .001, partial *η* ^2^ = 0.534, had significant main effects. Participants in the first person condition (*M* = 3.24, *SD* = 1.04) used more categories than those in the third person condition (*M* = 2.78, *SD* = 1.36). Participants in the guided condition (*M* = 3.89, *SD* = 0.57) used more categories than those in the open condition (*M* = 2.13, *SD* = 1.06). The interaction between the conditions was not significant, *F*(1, 106) = 1.622, *p* = .206, partial *η* ^2^ = 0.015.

Finally, only perspective, *F*(1, 106) = 4.858, *p* = .030, partial *η* ^2^ = 0.044, had a significant effect on the difficulty that participants reported having in imagining their FVS. Participants in the first person condition (*M* = 4.56, *SD* = 1.89) reported that it was easier to imagine their future self than participants in the third person condition (*M* = 3.75, *SD* = 1.094). Neither the guided versus open condition, *F*(1, 106) = 0.392, *p* = .533, partial *η* ^2^ = 0.004, nor the interaction between conditions, *F*(1, 106) = 1.692, *p* = .196, partial *η* ^2^ = 0.016, had a significant effect on the difficulty of imagining a FVS.

Thus, these results indicate that the guided manipulation resulted in more elaborate FVS (more words written and categories used). The first‐person perspective made it easier for participants to imagine their FVS.

5.4

*Profiles of FVS imagined per condition*

**Figure 1:**
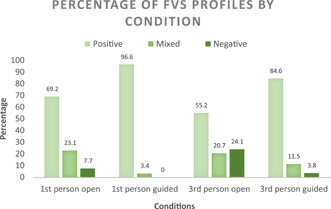
Percentage of positive, mixed, and negative future‐vaccinated selves (FVS) profiles expressed by participants in each condition.

We wanted to see if the conditions would lead to people expressing more or less positive FVS using the three profiles developed from the qualitative analysis. To do this we conducted a *χ* ^2^ test with the conditions as columns and the profiles as rows (note that for guided first‐person condition, although the actual cell value was 0 for negative profiles, we changed it to 1, to be able to calculate the *χ* ^2^). The profiles reported did differ by condition, *χ* ^2^ = 16.44, *p* = .012. An examination of the frequencies presented in Figure 1 shows that fewer negative or mixed profiles were imagined in the conditions that were guided. Thus, it seems that guiding participants to think about specific elements of their FVS led them to list more positive elements.

5.5

*Effects of experimental conditions on attitudes toward the COVID‐19 vaccine*

**Figure 2:**
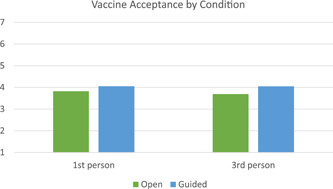
Average vaccine acceptance by condition.

**Figure 3:**
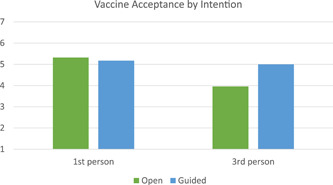
Average vaccine intention by condition.

To test the effect of our experimental conditions on the intention to receive the COVID‐19 vaccine and acceptance of the vaccine variables, we conducted a 2 × 2 multivariate analysis of variance (MANOVA). We ran the analysis with 5000 bootstraps. We found that there was a tendential main effect of both perspective, Wilks's lambda = 0.955, *F*(2, 105) = 2.448, *p* = .091, partial *η* ^2^ = 0.045, and guided versus open independent variable, Wilks's lambda = 0.948, *F*(2, 105) = 2.871, *p* = .061, partial *η* ^2^ = 0.052. The interaction was not significant, *p* = .247. Based on the result of the MANOVA we ran two follow‐ups 2 × 2 ANOVAs on intention and acceptance separately. For acceptance, there was only a main effect of the guided versus open condition, *F*(1, 106) = 5.720, *p* = .019, partial *η* ^2^ = 0.051. Individuals in the guided response condition had higher acceptance of the COVID‐19 vaccine than individuals in the open response condition, see Figure 2 for an illustration. For intention, there was only a main effect of perspective, *F*(1, 106) = 4.248, *p* = .042, partial *η* ^2^ = 0.039. People in the first‐person condition were more likely to intend to get the COVID‐19 vaccine, see Figure 3. Cell means from the follow‐up ANOVAs can be found in Table 4.

**Table 4:** Estimated marginal cell means and standard errors for COVID‐19 acceptance and intention by condition

	Perspective	Open versus guided		
		**Open**	**Guided**	**Total**
Acceptance				
	1st person	**3.821** (0.126)	**4.052** (0.119)	**3.963** (0.087)
	3rd person	**3.690** (0.119)	**4.045** (0.126)	**3.867** (0.087)
	Total	**3.755** (0.087)	**4.048** (0.087)	
Intention				
	1st person	**5.317** (0.381)	**5.172** (0.361)	**5.245** (0.262)
	3rd person	**3.96** (0.361)	**5.000** (0.381)	**4.480** (0.262)
	Total	**4.639** (0.262)	**5.068** (0.262)	

*Note*: Means are in bold and standard errors are in parentheses.

### 5.6 Discussion

In this study, we manipulated the ease with which participants could imagine their future selves by guiding them or leaving their responses open and perspective (first vs. third) on the efficacy of imagining a FVS. We found that participants in the guided condition were more likely to report solely positive FVS and reported greater acceptance of the COVID‐19 vaccine. We also found that participants in the first person condition reported higher intention to receive the COVID‐19 vaccine than those in the third person condition. In general, the categories of FVS were similar to those reported in Study 1, suggesting that we have captured the most frequent FVS in both studies.

## 6 GENERAL DISCUSSION

With this study we aimed to understand how people imagined their FVS and the effect that these future selves could have on their attitudes toward the COVID‐19 vaccine. In Study 1 we explored what factors might motivate low‐risk individuals to be vaccinated by asking participants to list five things that they imagined would be true for their FVS. From these responses we generated several categories of FVS including a return to social life, a resumption of leisure activities, a reduction in negative emotions, fewer restrictions related to COVID‐19, positive societal changes (e.g., greater focus on sustainability), worries about the side‐effects and effectiveness of the vaccine, fears about negative societal evolutions and feelings that nothing would change for their future self. We found that the imagined FVS tended to be positive, although a small number of participants reported some negative visions of the future. Finally, we found that the more difficult it was for participants to imagine their FVS the less likely they were to intend to receive the vaccine.

In Study 2, we found that participants who were in the guided condition used a larger number of words to describe their FVS and had higher levels of vaccine acceptance than those in the open condition. We also noted that guiding participants' responses in how they envisioned their FVS led to more positive responses than when participants could respond in any way they wished. We found that participants in the first person condition found imagining their FVS easier and expressed greater intentions to receive the COVID‐19 vaccine than those in the third person condition. Notably, in both Studies 1 and 2 participants reported similar categories of possible FVS.

### 6.1 Positive FVS as motivators of vaccination

Our study sheds light on what people consider the most important aspects of their FVS and thus the motivational factors that can promote vaccine acceptance and uptake. The most frequently cited FVS concerned the return of social activities such as seeing friends and family and returning to face‐to‐face studies at university. This result speaks to the isolation and loneliness that many felt during the pandemic due to decreased opportunities for social interaction related to governmental restrictions on movement and social contact (Killgore et al., 2020). Thus, returning to a normal social life seems to be a strong motivator of the intention to receive the COVID‐19 vaccine.

The second most frequent category was related to sports and leisure activities. Although they may seem frivolous or less important than other activities, leisure activities provide an important opportunity for young adults to explore their identity and make social connections (Layland et al., 2018) at a time when they are still forming their identities and social networks. Thus, the return to leisure activities may be an important motivator for this specific population in which most of our participants fell.

The third most frequently cited category related to having fewer negative emotions about COVID‐19 and this included fears of both oneself falling ill and causing one's family or friends to fall ill. For young people, vaccination seems to have prosocial motivations which is in line with studies on vaccine uptake showing that highlighting the prosocial benefits of vaccination (i.e., herd immunity and not infecting others) can increase the intention to receive vaccines for those who are at low risk (Jung & Albarracín, 2021; Tavolacci et al., 2021).

The final positive category related to the inconveniences of specific constraints related to COVID‐19 such as wearing a mask when in public spaces or respecting a curfew. Interestingly, in Study 2, this category became more frequently reported, perhaps because at the time of the study France had been under a strict curfew for 5 months and residents were required to return to their homes before 6 p.m.

### 6.2 Negative FVS as barriers to vaccination

While most FVS were positive, some participants imagined the negative consequences of being vaccinated. The fifth most common FVS related to worries about the COVID‐19 vaccine, both in terms of its safety and efficacy. This finding is congruent with studies of other vaccines in which researchers found concerns about the safety of vaccines protecting against diseases such as human papillomavirus (Conroy et al., 2009) and A(H1N1) (Galarce et al., 2011) to be a barrier to vaccination. Indeed, in April 2020, a survey of more than 5000 French people identified the belief that the COVID‐19 vaccine was not safe as the main cause for refusing vaccination, the second being a distrust of vaccines in general (Alleaume et al., 2021). The frequency of responses in which participants' anticipated negative effects from vaccination highlights the need for communication from public health officials about the efficacy and safety of the COVID‐19 vaccine, so that people can envision a FVS without being concerned about its side effects. For instance, presenting the very high efficacy rate of the COVID‐19 vaccine compared to the better‐known annual flu vaccine seems to be an effective strategy for convincing hesitant individuals (Davis et al., 2021).

Additionally, a smaller number of participants worried about the negative societal consequences of the COVID‐19 vaccine, such as the fear that the government may take advantage of the end of the health crisis to impose lasting restrictions and an even more unequal society. This concern may reflect that being less trusting of government is related to vaccine hesitancy (Salmon et al., 2005) and a lack of institutional trust has been linked to engaging in fewer COVID‐19 prevention behaviors (Caplanova et al., 2021), and hostility towards the new vaccine (Bajos et al., 2022; Van Oost et al., 2022). For French youths in particular, lack of trust in the government's management of the pandemic was found to predict vaccine acceptance and intention to vaccinate, both directly and through an increase in perceived stress (Brun et al., 2022).

### 6.3 Differences between vaccine acceptance and intention

One interesting finding from Study 2 was that COVID‐19 vaccine acceptance and intentions to receive the vaccine were each only influenced by one of our independent variables. Vaccine acceptance was higher for those participants in the guided condition compared to the open condition. Participants in the guided condition also produced more elaborate descriptions of their future selves, using both more words and categories. This extra elaboration may have activated increased cognition about the vaccine as more elaborate future selves tend to increase cognitions about the future (Markus & Nurius, 1986). Research on health messaging and preventive health behaviors highlights the fact that increased cognition about health behaviors is more effective at changing attitudes than behaviors (Dunlop et al., 2010).

Conversely, activating emotion, rather than cognition, is linked to actual behavior change (Conner et al., 2011; Keer et al., 2013). This may be why our perspective manipulation influenced behavior, where imagining a FVS from the first‐person perspective was associated with greater behavioral intention to receive the vaccine. Indeed, numerous studies in the field of autobiographical memory show that recalling personal memories from the first‐person perspective leads to more intense emotions compared to memories remembered in the third person (Holmes & Mathews, 2010). This is true also when imagining hypothetical situations: people asked to visualize positive events in the first person display a more enhanced mood than people who see them only as an observer (Holmes et al., 2008).

In a study examining the difference between emotional and cognitive pathways to health behaviors, Keer et al. (2010) found that activating cognitions about health led to changes in how health behaviors were evaluated, but it was only this change in attitudes that then led to behavioral changes. These previous results in the health literature align with our findings. If participants had increased cognitions about their future lives in the guided condition, this may have worked to change how they evaluated the COVID‐19 vaccine and thus increased the positivity of their attitudes concerning the vaccine's safety. However, this change in attitudes may not have been strong enough to increase behavioral intention, as we did not see any influence of the guided condition on intention to receive the COVID‐19 vaccine. However, imagining a positive future in the first person may have activated more positive emotions and led to behavioral intentions to receive the vaccine.

## 7 LIMITATIONS AND FUTURE DIRECTIONS

While our study does provide strong evidence for the motivational power of FVS, it has several limitations. One limitation concerns the experimental manipulations in Study 2. While we instructed participants to imagine their FVS in first or third person, we have no way to verify that they did take the perspective suggested. Although participants reported greater difficulty on average in the third‐person conditions, we cannot be sure that they followed the instructions. In future studies, a manipulation check that would check participants' understanding of the instructions would be a first step (e.g., Libby et al., 2014) and participants could be asked explicitly to write in first or third person. Additionally, we supposed that the differences found in the influence of first versus third person future selves on vaccination intention could be due to their different emotional power, with the first‐person perspective eliciting a stronger emotional response. To verify this hypothesis, future studies should seek to measure emotions immediately after the mental imagery task.

Finally, we did not measure participants' trust in government or political affiliation. In France, attitudes towards the COVID‐19 vaccine and intention to get vaccinated did not follow a traditional political pattern (left vs. right) but rather divided people who felt close to governing parties and those who identified with the extremes—whether on the left or the right of the political spectrum, or who did not support any party (Ward et al., 2020). These political divides also influence French public opinion on mandatory vaccination, with partisans of the far left for instance being more likely to oppose mandatory COVID‐19 vaccine than partisans of the centrist party in power (Gagneux‐Brunon et al., 2022). Having voted for an anti‐establishment candidate at the 2017 presidential election was also a predictor of the refusal to get vaccinated, both before and after the vaccine mandate (Débarre et al., 2022). Thus, future studies should take into account these variables as possible moderators of the effectiveness of a prevention campaign based on FVS.

## 8 CONCLUSION

Our research shows that health information campaigns that help people think about the future benefits of vaccination could help increase COVID‐19 vaccination rates. The novelty of our study is our consideration of other factors than risk and prosocial motivations that may motivate vaccine acceptance and uptake. As our study shows, people are motivated by considering their future selves engaging in a wide range of situations including socializing, leisure activities, and experiencing reduced negative emotions related to COVID‐19. Campaigners could include a variety of these categories in their attempts to motivate vaccination. Indeed, while the television advertisement cited in the first paragraph of this article is a first step in the right direction, campaigners should also include FVS that appeal to a wide range of individuals with varied interests and diffuse these advertisements on social media, a key information source for young people especially during the pandemic (e.g., Qiao et al., 2022). These campaigns should present positive FVS that are attainable and evoke emotion to have the most impact. As our study shows, imagining a specific future self has the motivational power to influence both acceptance of the COVID‐19 vaccine and intention to receive the vaccine when the future envisioned is positive, elaborate, and easy for the individual to imagine.

## CONFLICT OF INTEREST

The authors declare no conflict of interest.

## ETHICS STATEMENT

Both studies were approved by the Northumbria University Ethics Committee, Study 1 approval number 28452, Study 2 approval number 29248.

## Data Availability

The quantitative data that support the findings of this study are openly available in OSF at https://osf.io/753k8/?view_only=6e0282da1fcf44688de45ae1e919cb27.
